# An evaluation of sucrose as a possible contaminant in e-liquids for electronic cigarettes by hydrophilic interaction liquid chromatography–tandem mass spectrometry

**DOI:** 10.1007/s00216-014-7690-2

**Published:** 2014-03-25

**Authors:** Paweł Kubica, Andrzej Wasik, Agata Kot-Wasik, Jacek Namieśnik

**Affiliations:** Department of Analytical Chemistry, Chemical Faculty, Gdańsk University of Technology, Narutowicza 11/12, 80-233 Gdańsk, Poland

**Keywords:** Electronic cigarettes, Hydrophilic interaction liquid chromatography-tandem mass spectrometry, Carbohydrates

## Abstract

The influence of sucrose combustion products on smoking and nicotine addiction is still controversial because the presence of the sucrose may be treated as a source of aldehydes and organic acids. In e-liquids used as refills for electronic cigarettes, which are made primarily of poly(propylene glycol), glycerine and ethanol, sucrose may be present at trace levels, and its impact on mainstream smoke formation, and hence on human health and smoking/nicotine addiction is unknown. An analytical method was developed where high-performance liquid chromatography in hydrophilic interaction liquid chromatography mode and tandem mass spectrometry were used for fast and simple determination of sucrose and other saccharides in e-liquids for electronic cigarettes. Minimal effort was required in the sample preparation step, and satisfactory results were obtained, and the sample matrix had an insignificant impact. The chromatographic separation was done using an Ascentis Express OH5 column (150 mm × 2.1 mm, 2.7 μm). The coefficients of variation for within-day precision for three concentrations were 2.4 %, 1.6 % and 2.3 %, and the between-day coefficients of variation for a single concentration were 2.1 %, 2.5 % and 1.7 % measured on the next 3 days. The detection limit was 0.73 μg/g, and the sucrose content in e-liquids ranged from 0.76 to 72.93 μg/g among 37 samples. Moreover, with the method presented it is possible to determine the presence of other saccharides such as fructose, glucose, maltose and lactose. However, only sucrose was found in all samples of e-liquids. The proposed method is rapid, simple and reliable in terms of high-performance liquid chromatography coupled with tandem mass spectrometry.

## Introduction

The use of electronic cigarettes (e-cigarettes) as an alternative to traditional tobacco smoking and as part of therapy in nicotine and smoking addiction is becoming more and more popular. In our previous research, we proved that not all liquids for e-cigarettes marked as nicotine-free are actually free of nicotine [1]. Sucrose is a popular additive in tobacco products and is commonly used in the production processes and to enhance the taste and flavour of the tobacco. Moreover, sucrose occurs naturally in tobacco leaves [2–4] and can be determined by liquid chromatography combined with mass spectrometry [5] or by other techniques [6]. The exact mechanism of the formation of aldehydes and organic acids during combustion of sucrose and other sugars in tobacco is still not fully understood [7, 8]. However, it is known that during this process organic acids and aldehydes may be formed [3, 9–12]. Aldehydes coming from sugars during the combustion of tobacco may have the reinforcing effect responsible for increased addiction to nicotine and smoking [9, 13]. The high temperature during the combustion of tobacco (from 600 to 900 °C during drawing) is responsible for the formation of aldehydes and organic acids. The working temperature of the heating element of e-cigarettes is variable owing to the cooling of inhaled air flow [14]. From one point of view, this temperature may seem too low (around 250 °C) to lead to the formation of aldehydes and organic acids, but there are reports [3, 15] indicating fairly quick formation of aldehydes from sucrose during cigarette smoking even at temperatures as low as 200 °C. Furthermore, more than ten different aldehydes were detected in e-cigarette aerosol [16]. Despite this, the temperature is high enough to evaporate the main ingredient, which is poly(propylene glycol), with a boiling temperature of around 188 °C. All of the e-liquids available on the market are based on poly(propylene glycol) (above 80 %), glycerine and even ethanol (from 5 to 15 %) according to the labels attached. What is more, none of the labels of e-liquids on the market state that the products contain sucrose or may contain it at a trace level.

The purpose of the project was to develop a rapid and simple method for the evaluation of sucrose content in e-liquids by hydrophilic interaction liquid chromatography (HILIC) and tandem mass spectrometry (MS/MS) with electrospray ionization and multiple reaction monitoring. The proposed analytical method allows the determination of the sucrose content in less than 4 min, with minimal effort required for sample preparation.

## Materials and methods

### Chemicals

Standards of sucrose, glucose, fructose, maltose, lactose and raffinose as well as ammonium formate were purchased from Sigma-Aldrich (St. Louis, USA). High-performance liquid chromatography (HPLC) grade acetonitrile was purchased from Merck (Darmstadt, Germany). Ultrapure water was obtained with the use of an HLP5 system from Hydrolab (Wiślina, Poland).

### Samples

Thirty-seven samples from different producers of popular e-cigarettes were purchased on the local market. The labels attached to the products did not contain any information about carbohydrate content.

### Preparation of standards and calibration solutions

Stock solutions of sucrose, glucose, fructose, maltose, lactose and raffinose (used as an internal standard) were prepared by dissolving an appropriate amount of each in a mixture of acetonitrile and water (80:20). The final concentration of each carbohydrate was 50 μg/mL. The purity of raffinose, with regard to the presence of sucrose, was tested, and no traces of sucrose were detected. All samples tested were raffinose-free, and therefore use of raffinose as an internal standard is acceptable. Calibration solutions were prepared by dilution of the stock solution with the mobile phase to obtain sucrose concentrations of 10, 50, 100, 150, 200 and 400 ng/mL, whereas the concentration of the internal standard was kept at 400 ng/mL. All solutions were stored in a refrigerator at 4 °C and every week new solutions were made.

### Sample preparation and preparation of fortified samples

Fifty milligrams of each sample was added to a 10-mL volumetric flask. Internal standard solution (40 μL) was added, and the flask was filled up to the mark with the acetonitrile–water (80:20) mixture. Fortified samples were prepared as follows: a randomly selected sample with sucrose content below the limit of detection (LOD) was spiked with the appropriate amount of sucrose standard to obtain 10, 20 and 30 μg/g solutions (these correspond to 50, 100 and 150 ng/mL after sample preparation). The fortified samples were used for repeatability and trueness (recovery) determination.

### MS/MS conditions

All analyses were performed with a Q-Trap 4000 triple-quadrupole mass spectrometer from Applied Biosystems (Foster City, CA, USA) with electrospray ionization in negative mode, using Analyst® 1.5.2. Analyte-specific multiple reaction monitoring conditions and ion source parameters were determined using an appropriate Analyst® feature, by the infusion of a 200 ng/mL solution of each carbohydrate, and in flow injection mode, respectively. Optimum detection conditions are presented in Table 1.

**Table 1:** Optimal parameters for the ion transitions monitored and tandem mass spectrometry (*MS*/*MS*) operational parameters

Parameters for the ion transitions monitored					
Analyte	Pseudomolecular ion→fragment ion	DP (V)	EP (V)	CXP (V)	CE (V)
Sucrose	341.0→179.1	-100	-10	-5	-20
	341.0→89.0^a^			-5	-26
Glucose	178.9→88.9	-75	-10	-13	-10
Fructose	178.9→88.9	-50	-10	-3	-12
Maltose/lactose	341.3→160.7	-80	-10	-17	-10
Raffinose	503.2→178.9	-125	-10	-13	-30
MS/MS operation parameters					
Curtain gas (psi)	Temperature (°C)	Nebulizer gas (psi)		Turbo gas (psi)	
20	550	50		40	

*DP* declustering potential, *EP* entrance potential, *CXP* collision cell exit potential, *CE* collision energy

^a^Qualitative transition

### HPLC conditions

**Fig. 1:**
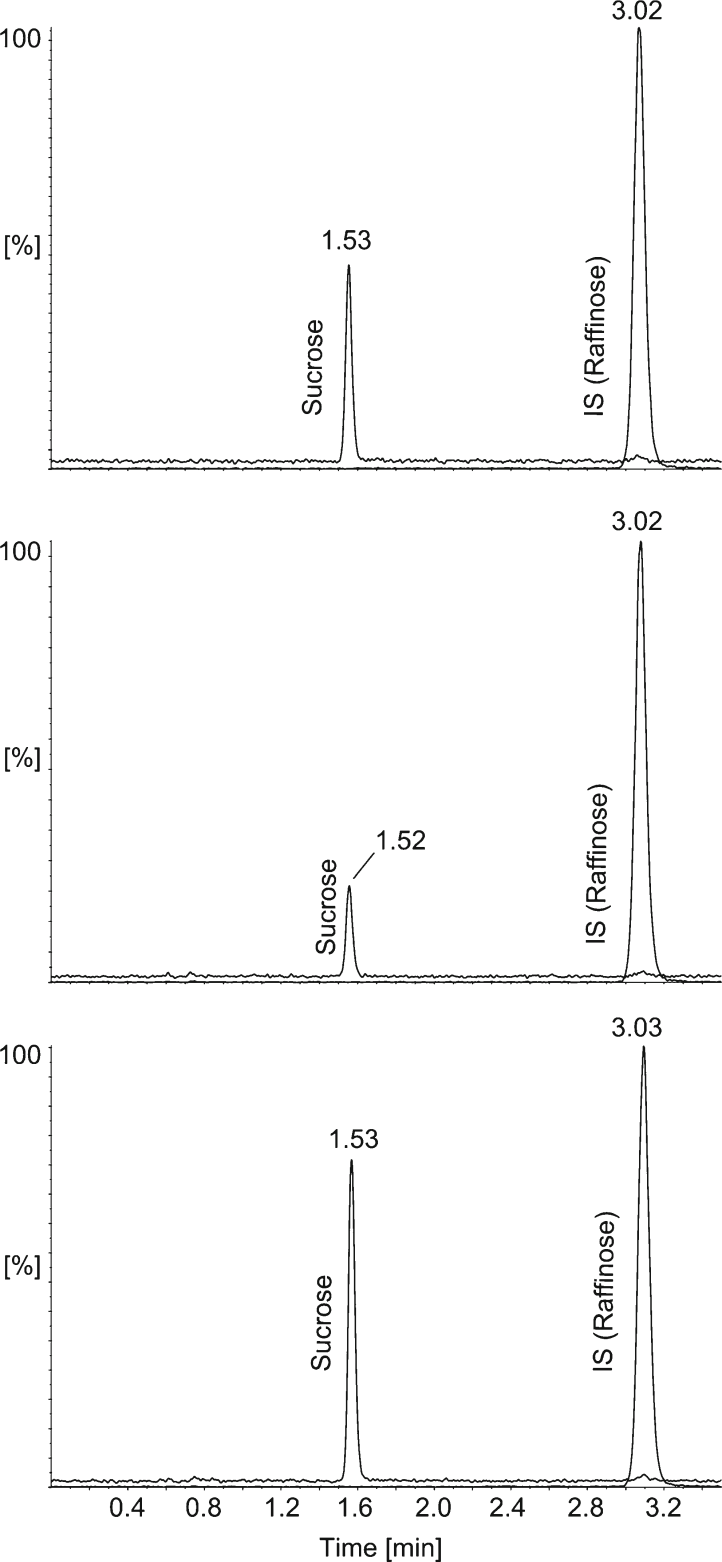
Examples of chromatograms obtained in hydrophilic interaction liquid chromatography (HILIC) mode. From the *top*: mixture of standards sucrose (100 ng/mL) and raffinose (400 ng/mL), chromatogram of real sample C cherry (sucrose concentration 8.00 μg/g), chromatogram of real sample D coffee (sucrose concentration 40.82 μg/g). *IS* internal standard

The chromatographic separation was performed using the HPLC–MS/MS system consisting of a pump, degasser, autosampler and column oven from the Agilent 1200 series. The separation of analytes was achieved in the HILIC mode using an Ascentis Express column (Supelco, Bellefonte, PA, USA; 150 mm × 2.1 mm, 2.7 μm, pore size 80 Å). The column oven temperature was set to 35 °C. The mobile phase contained acetonitrile (solvent A) and ammonium formate buffer (solvent B) adjusted to pH 6.8. The presence of ammonium formate is necessary to obtain narrow and symmetrical peaks. Too high a concentration of buffer will suppress the detector signal, and therefore its concentration was kept to a minimum, i.e. 2 mM. The separation of sugars was performed in the isocratic mode (80 % solvent A and 20 % solvent B) at a flow rate of 0.7 mL/min. The injection volume was set to 5 μL. The total time of the chromatographic run was 3.5 min. Chromatograms of standards (sucrose and raffinose) and chromatograms of selected real samples are presented in Fig. 1.

## Results and discussion

### The possible presence of other sugars in e-liquids

**Fig. 2:**
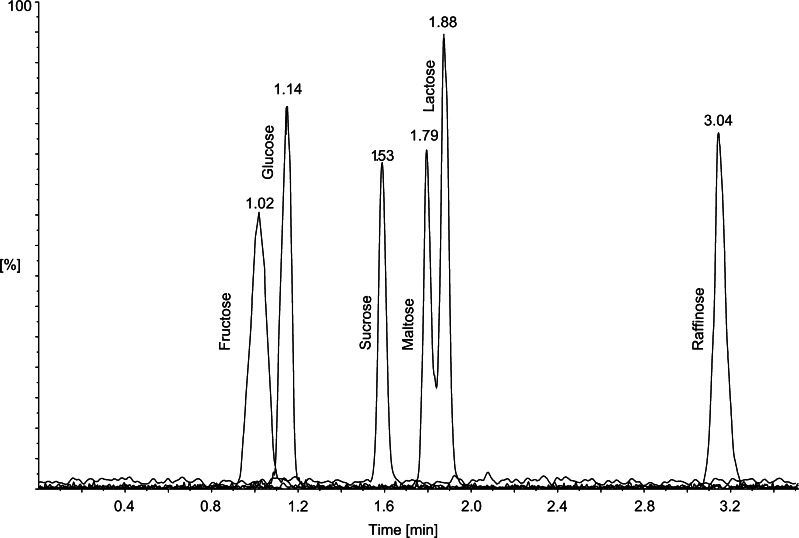
Chromatogram of a mixture of analytes: fructose, glucose, sucrose, maltose, lactose and raffinose (each at 200 ng/mL) detected by negative electrospray ionization tandem mass spectrometry in HILIC mode

Most of the disaccharides are built of monomers such as glucose, galactose and fructose. This means that they will generate the same pseudomolecular and fragmentation ions during MS/MS. In fact, only the difference in the retention times between different sugars allows their identification. The samples of e-liquids were tested for the presence of two disaccharides (lactose and maltose) and two common monosaccharides (fructose and glucose) in order to exclude their possible coelution with sucrose. The mixture containing each sugar at 200 ng/mL was analysed under the conditions described in “HPLC conditions”. The chromatogram presenting the separation of standards is shown in Fig. 2. Under the proposed HPLC conditions, sucrose is baseline-separated from other sugars; hence, they will not interfere with its quantitative determination. None of the 37 samples analysed were found to contain sugars other than sucrose.

### Within-laboratory validation

#### Analytical figures of merit

A six-point calibration curve was constructed using raffinose as an internal standard, and each calibration solution (see “Preparation of standards and calibration solutions”) was analysed in triplicate. The curve was linear in the range of concentrations studied. The LOD was calculated with the equation LOD = 3.3*S*_b_/*a*, where *S*_b_ is the standard deviation of the intercept and *a* is the slope of the calibration curve. The limit of quantitation was calculated as three times the LOD. The LOD obtained (0.73 μg/g) is similar to [17, 18] or even lower [19, 20] than the values reported by others. Within-day precision was estimated by replicate (*n* = 6) analysis of samples fortified at three concentrations (10, 20 and 30 μg/g) on 1 day. Data obtained during the within-day precision investigation were also used to assess the trueness of the method. Intermediate (between-day) precision was verified by analysing the single fortified solution (20 μg/g) for three consecutive days. Again, each analysis was performed six times (*n* = 6).

As can be seen from Table 2, the recovery values at all spiking levels are close to 100 %, which means that no matrix effects or bias was observed. This allows the use of external calibration instead of a matrix-matched approach. The method also performs well in terms of precision. In no case was the coefficient of variation greater than 2.5 %.

**Table 2:** Determination of sucrose in fortified e-liquid samples: calibration parameters, trueness and repeatability data

Analyte	Calibration curve equation (6 point, *n* = 3)	*S*_a_^a^	*S*_b_^c^	*R* ^2d^	LOD (μg/g)	LOQ (μg/g)
Sucrose	*y* = 0.001746*x* + 0.0479	0.000011	0.0019	0.9995	0.73	2.2
	Within-day precision (3 spiking levels, *n* = 6)					
	Spiking level (μg/g)	Recovery^b^ (%)	CV (%)			
	10	101.4	2.4			
	20	101.1	1.6			
	30	98.3	2.3			
	Between-day precision (1 spiking level, 20 μg/g, *n* = 6)					
	Day	Recovery^b^ (%)	CV (%)			
	1	99.6	2.1			
	2	105.8	2.5			
	3	102.9	1.7			

*LOD* limit of detection, *LOQ* limit of quantitation, *SD* standard deviation, *CV* coefficient of variation

^a^Standard deviation of the slope

^b^Calculated as the ratio between the mean concentration found from the calibration curve and the spiking level

^c^Standard deviation of the constant term

^d^Coefficient of determination

#### Analysis of real samples

All samples were prepared according to the protocol described in “Sample preparation and preparation of fortified samples”. Samples were chosen from among the most manufacturers and the popular brands available on the market. The content of sucrose in the samples analysed is presented in Table 3.

**Table 3:** Concentration of sucrose in e-liquids for electronic cigarettes: analysis of real samples

Company	Taste	Concentration of sucrose ± SD (μg/g)
A	Black	1.11 ± 0.21
	Cherry	2.28 ± 0.11
	Menthol	23.73 ± 0.81
B	Camel	0.68^a^
	Chocolate	7.315 ± 0.095
	Grape	5.04 ± 0.28
	Orange	0.56^a^
	Strawberry	0.64^a^
	Watermelon	1.99 ± 0.11
C	Banana	2.31 ± 0.19
	Camel	10.89 ± 0.14
	Cherry	8.006 ± 0.024
	Fruit mix	5.62 ± 0.37
	Marlboro	20.15 ± 0.43
	Menthol	10.66 ± 0.55
	L&M	0.784 ± 0.053
	Red Bull	19.06 ± 0.34
	Vanilla	1.027 ± 0.093
D	Apple	25.07 ± 0.35
	Camel	1.211 ± 0.052
	Cherry	8.65 ± 0.36
	Chocolate	72.93 ± 0.72
	Coffee	40.82 ± 0.52
	L&M	9.86 ± 0.28
	Marlboro	13.11 ± 0.20
	Red Bull	13.46 ± 0.23
	Strawberry	8.22 ± 0.14
	Tobacco	19.91 ± 0.54
E	Cherry	0.62^a^
	Coffee	3.40 ± 0.20
F	Coffee	0.76 ± 0.12
	Menthol	26.23 ± 0.29
	Red Bull	7.74 ± 0.20
	Tea	22.25 ± 0.46
	Tobacco	29.82 ± 0.43
G	Fruit mix	1.80 ± 0.13
	Menthol	11.67 ± 0.35

^a^Informative value only, samples were reanalysed with less dilution (see the text for details)

Only in four samples was the sucrose content below the LOD or *C*_min_ used for the construction of the calibration curve. These samples were reanalysed with less dilution (5 mL instead of 10 mL). There is no clear relationship between the sucrose content and the manufacturer. Among the samples of a given brand, one will find e-liquids that are almost sucrose-free together with others high in sucrose. For example, most of the samples from producer B are low in sucrose (less than 1 μg/g), but there is an exception. Chocolate-flavoured e-liquid was found to contain sucrose at a concentration of 7.3 μg/g. The opposite situation can be observed in the case of producers C and D. Here, most of the samples were found to be high in sucrose, but there were a few exceptions. Likewise, no direct relationship was found between the flavour and the sucrose content. Only in the case of menthol-flavoured e-liquids was the sucrose content rather high in each sample.

## Conclusions

The purpose of this project was to develop a simple, reliable and sensitive method for the determination of sucrose in e-liquids with minimum effort for sample preparation. The method developed may be helpful in future research on e-cigarettes. The main advantages of the method presented are the low LOD (0.73 μg/g) and the short analysis time, without the need to stabilize the column owing to the isocratic separation mode. The source of sucrose in e-liquids is unknown. One of the possibilities is that sucrose is a component of the flavour/taste additives, or it is a contaminant from the production process. Another possibility is that sucrose is extracted along with nicotine from tobacco leaves, although in such a case reducing sugars such as fructose and glucose should also be present. Still, there is much controversy about the safety of e-cigarettes, and the method developed may be helpful in quality control, or research on the impact of e-liquid content and the composition of e-cigarette aerosol.
